# Insights into measuring health disparities using electronic health records from a statewide network of health systems: A case study

**DOI:** 10.1017/cts.2022.521

**Published:** 2023-02-01

**Authors:** Maureen A. Smith, Matthew Gigot, Abbey Harburn, Lauren Bednarz, Katherine Curtis, Jomol Mathew, Dorothy Farrar-Edwards

**Affiliations:** 1 Department of Population Health Sciences, University of Wisconsin School of Medicine and Public Health, Madison, WI, USA; 2 Department of Family Medicine and Community Health, University of Wisconsin School of Medicine and Public Health, Madison, WI, USA; 3 Health Innovation Program, University of Wisconsin School of Medicine and Public Health, Madison, WI, USA; 4 Wisconsin Collaborative for Healthcare Quality, Madison, WI, USA; 5 Department of Community and Environmental Sociology, University of Wisconsin-Madison, Madison, WI, USA; 6 Departments of Kinesiology and Medicine, University of Wisconsin-Madison, Madison, WI, USA

**Keywords:** Health equity, electronic health records, health disparities, quality improvement collaboratives, public reporting, learning health systems, social determinants of health

## Abstract

Within Wisconsin, our residents experience some of the worst health disparities in the nation. Public reporting on disparities in the quality of care is important to achieving accountability for reducing disparities over time and has been associated with improvements in care. Disparities reporting using statewide electronic health records (EHR) data would allow efficient and regular reporting, but there are significant challenges with missing data and data harmonization. We report our experience in creating a statewide, centralized EHR data repository to support health systems in reducing health disparities through public reporting. We partnered with the Wisconsin Collaborative for Healthcare Quality (the “Collaborative”), which houses patient-level EHR data from 25 health systems including validated metrics of healthcare quality. We undertook a detailed assessment of potential disparity indicators (race and ethnicity, insurance status and type, and geographic disparity). Challenges for each indicator are described, with solutions encompassing internal (health system) harmonization, central (Collaborative) harmonization, and centralized data processing. Key lessons include engaging health systems in identifying disparity indicators, aligning with system priorities, measuring indicators already collected in the EHR to minimize burden, and facilitating workgroups with health systems to build relationships, improve data collection, and develop initiatives to address disparities in healthcare.

## Introduction

Within Wisconsin, our residents experience some of the worst health disparities in the nation. In 2021, Wisconsin ranked 32nd of 38 states in an overall health ranking for Black people with similar results for Latinx/Hispanic people [1]. Nationally and within the state, disparities in healthcare exist across multiple determinants of health including race and ethnicity [2], socioeconomic status, health insurance coverage, and geography (rural and urban) [3]. Public reporting on disparities in the quality of care is an important step to achieving accountability for reducing disparities over time [4] and has been associated with improvements in care [5]. Already a leader in public reporting of the quality of care [5–7], the Wisconsin Collaborative for Healthcare Quality (the “Collaborative”) is now considering publicly reporting quality measures separately for populations experiencing health disparities. Beginning in 2003, the Collaborative created a regional data repository with EHR data from 25 health systems that includes validated metrics on the quality of healthcare. Because the Collaborative publicly reports on health system quality twice each year, there is a significant opportunity to enhance this regular reporting with reports on disparities in the quality of care.

However, data on disparity indicators (i.e., race, ethnicity, and language) are often not available, not complete, or not completely reliable [8]. Public reporting on disparities will require addressing these substantial challenges in obtaining and using key data fields [9]. Data harmonization involves integrating disparate data of varying types, sources, and formats across many health systems to improve the quality, reusability, and interoperability of data [10]. Despite widespread hopes that electronic health records (EHRs) would streamline quality measurement [11], statewide efforts to harmonize EHR data have struggled to use the data to construct quality measures [12,13]. The Collaborative has substantial expertise in this harmonization process for EHR data but had not previously undertaken data harmonization for disparities indicators. To assess feasibility and design a solution to achieve a statewide system of public reporting on disparities in the quality of care, the Collaborative partnered with the University of Wisconsin-Madison (UW) Health Innovation Program, an affiliate of the UW Institute for Clinical and Translational Research.

We report here the experience of the Collaborative in assessing the feasibility of creating a statewide, centralized EHR data repository to support health systems in measuring, monitoring, and reducing health disparities. We describe our detailed assessment on the availability and quality of potential disparity indicators in the Collaborative data repository. We identify challenges for the use of each indicator and describe our solutions to health disparities measurement and monitoring at a statewide level.

## Methods

### Population

In 2003, the Collaborative established a methodology to create and share public reports of quality measures based on data extracted directly from the EHRs of health systems [7]. The Collaborative data repository includes patient-level data from 25 health systems, representing approximately 65% of the primary care providers and their patients in the state. Key elements required for metrics construction are validated, audited, and maintained within each health system and submitted to the repository (e.g., immunizations). The repository has recently transitioned to a cloud-based data analytics platform. Three additional health systems continue to construct quality measures internally and report only aggregated information to the Collaborative, with plans to transition to the cloud-based system in the future.

### Data

The Collaborative uses a common data model across all health systems using a custom relational database structure that was developed by the Collaborative in 2003 and enhanced over the years. Structured patient-level data are submitted to the repository on demographics, encounters, hospitalizations, medications, laboratory values, clinical values (e.g., blood pressures), problems, smoking history, providers, and clinics. Some health systems submit data using the common data model while others submit data using other mechanisms (e.g., HL7 messages). For these health systems, data are harmonized to this common data model. For most health systems, the data are reported at an extremely granular level (e.g., there are hundreds of distinct values for race, ethnicity, and language).

The data are evaluated at multiple stages in the submission and aggregation process for completeness, consistency, valid content, and referential integrity. Historically, data that are not used for construction of quality metrics (e.g., disparity indicators) have not undergone the same level of quality checks.

Health systems provide a separate mapping that assigns each data value to one of the Collaborative’s standard categories. These mappings are maintained and updated at least every six months. The Collaborative applies the mappings to the values to create the Collaborative’s standard categories for use in constructing quality metrics and also retains the original values in the data repository. As a result, for many but not all health systems, additional detail is available beyond the standard categorizations.

The Collaborative’s data are stored in a cloud-based repository with restricted access. The primary users of the data are the health systems themselves, for public reporting and quality improvement purposes. Access to the data for research purposes is managed through a partnership with the UW Health Innovation Program, which maintains a copy of the data in SAS format in a secure environment with computing resources and statistical software. Data are accessed and analyzed on a secure virtual machine after project approval, completion of human subjects (IRB) and data use agreements, and development of the project dataset. A data services fee is applied based on the scope of the data and programming effort required. In rare cases, deidentified data can be made available outside the secure environment with appropriate approvals. Interested researchers can contact dataservices@hip.wisc.edu for more information on using these data for research purposes.

### Quality Reporting at the Collaborative

Using the data repository, over 25 quality metrics at the population, health system, and clinic levels are calculated and reported on a public-facing website twice each year for over 4,000,000 patients in the state of Wisconsin (https://reports.wchq.org/statewide-results). These metrics are constructed from the structured patient-level data submitted to the repository. After construction, the metrics are maintained in the repository at the patient level. These metrics focus on cancer screening (breast, cervical, colorectal); screening for clinical depression; blood pressure control; pediatrics (adolescent immunization, childhood immunization, human papillomavirus vaccine (HPV), well child visit first 15 months of life); diabetes (blood pressure control, blood sugar control, tobacco-free, blood sugar testing, daily aspirin, statin use, eGFR testing, kidney function monitoring); and ischemic vascular disease (blood pressure control, tobacco-free, daily aspirin, statin use). Many other quality metrics are constructed and used for internal reporting to health systems but not reported on the public-facing website.

### Disparity Indicators

In 2018, the Collaborative launched a project with the UW Health Innovation Program to identify disparity indicators within the data repository to support the public reporting of health disparity measures. Our approach builds on the National Quality Forum (NQF) “Roadmap for Promoting Health Equity and Reducing Disparities” to identify and prioritize disparities through the stratification of performance measures [14]. This project was deemed not research and therefore exempt from IRB oversight at the University of Wisconsin-Madison.

Engagement of health system stakeholders was critical to obtaining buy-in to disparities measurement. Using the standard engagement process that supports the development of any new Collaborative quality measure, the Collaborative’s Measurement Advisory Committee conducted individual interviews with its participating health systems to identify possible disparity indicators from the NQF Roadmap and categorize them based on health system interest and availability (Table 1). Based on this process, the Collaborative proposed focusing on several currently available indicators to stratify existing quality of care measures: race and ethnicity, insurance status and type, and geographic disparity. The selection process prioritized indicators that health systems were already collecting due to feasibility and health system buy-in, significantly reducing health system burden.

**Table 1.** Disparity indicators and their level of interest to health systems

Factor/Concept
**Available in Health System Data**
Race/Ethnicity
Insurance status
Medicaid status
No insurance
Geographic disparity
Marital status
**Not available, but of interest to Health Systems**
Education
Living alone
Employment status
Health literacy
**Not available, of less interest to Health Systems**
Income
Income in relation to federal poverty level
Household income
Social security supplemental income
Homelessness
Housing instability
English proficiency
Contextual-proportion vacant housing
Contextual-crime rate
Social support
Occupation
Literacy
Local/state funding for safety net providers

### Race and Ethnicity

The Collaborative health systems prioritized measuring disparities in care for racial and ethnic minorities as these individuals experience substantial gaps in the quality of healthcare [15]. Race and ethnicity data are typically collected in the EHR, and many organizations use an extensive standardized list of racial/ethnic categories provided by their EHR vendor. The Collaborative maps these categories to the definitions provided by the Office of Management and Budget (OMB). The OMB minimum categories for race are American Indian or Alaska Native, Asian, Black or African American, Native Hawaiian or Other Pacific Islander, and White. The OMB minimum categories for ethnicity are Hispanic or Latino and Not Hispanic or Latino.

### Insurance Status and Type

Insurance status was of particular interest as a disparity indicator to identify individuals who were uninsured or covered by Medicaid. Disparities in healthcare quality by insurance status (insured versus uninsured) and insurance type (e.g., public versus private, Medicaid) have been well documented [16–18]. Insurance status and type is required for payment of health system activities by health insurers, ensuring collection in the EHR by all Collaborative health systems. Insurance information is mapped to standard categories including Commercial, Medicaid, Medicare fee-for-service, Medicare Accountable Care Organization, Other Medicare (e.g., Medicare Advantage), and Uninsured.

### Geographic Disparity

The Collaborative also recognized the need to map disparities by geography, given the wide range of both rural and urban areas in Wisconsin. Geography including rural-urban residence and neighborhood disadvantage has been linked to disparities in the quality of care [19,20]. Our prior work in building a rural-urban geodisparity model has shown that disparities exist within Wisconsin in both rural and urban underserved areas [21], and that, at a national level, neighborhood disadvantage is strongly related to rehospitalization rates [22]. Both ZIP code (needed for rural-urban geodisparity) and address (needed for neighborhood disadvantage) are collected by the Collaborative health systems in their EHRs, but only ZIP code was available in the Collaborative data repository.

## Results

### Quality Assessment and Implementation Plan

**Fig. 1.**
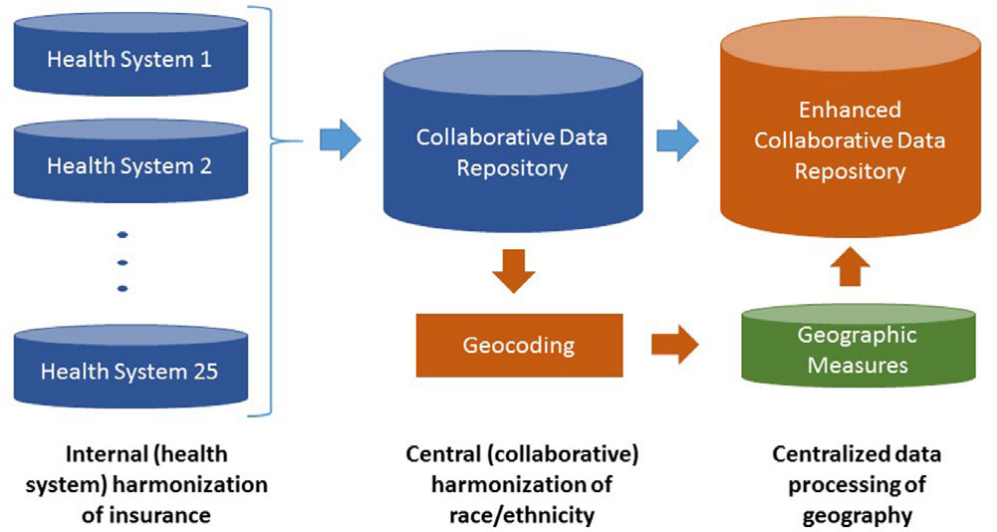
Implementation plan for health equity indicators in the Collaborative data repository.

We undertook a detailed assessment of the proposed disparity indicators within the Collaborative data repository. Because the Collaborative has historically collected demographic and insurance data, but never used it for reporting, the quality of data was not at the same level as other data being submitted that is regularly used for reporting. Challenges for each indicator are described along with proposed solutions that will be incorporated into our implementation plan for enhancing the Collaborative data repository (Fig. 1). A key criterion for any solution was minimizing the burden placed on the staff of each health system. The Collaborative has developed a best practices guide for submission of these disparity indicators that will be available at www.hipxchange.org/DisparityIndicators.

### Race and Ethnicity

All health systems submit race and ethnicity data to the data repository, thereby representing a low burden for data collection. Many organizations use a comprehensive list of racial/ethnic categories, although a few health systems had missing information. The missing data challenge for the race/ethnicity fields was the result of patients not being asked and/or patients declining to answer. The Collaborative staff worked with these health systems to reduce the level of missing information. Using the data and value descriptions submitted by each organization, Collaborative staff were able to successfully harmonize race and ethnicity from each organization to a standard set of categories. By harmonizing at the level of the Collaborative data repository (“central harmonization”) rather than within the health system (“internal harmonization”), the burden on the health system was low.

Insurance Status and Type. Twenty-three of the 25 health systems submitted insurance information harmonized to a standard set of categories (Commercial, Medicaid, Medicare, and Uninsured). The Collaborative staff worked with the remaining two systems to submit insurance status and type. However, a significant harmonization challenge was identified. Upon discussion with data submitters, it was identified that many systems using the Epic EHR were collecting insurance information using the financial status field. Unless the health system develops a separate internal harmonization process, this field will frequently map Medicaid and Medicare health maintenance organization (HMO) providers to the Commercial category as the insurance is provided by a commercial insurance company. Given the nuances of multiple insurance products, it was determined that each health system would need to harmonize their Medicare and Medicaid HMO products to the standard categorization (internal harmonization).

Geographic Disparity. Five-digit ZIP code is needed to construct rural-urban geodisparity, while residential address is needed to map patients to census tracts and construct neighborhood disadvantage measures. All of the health systems submit ZIP code information to the Collaborative data repository but none currently submit residential address information. As a result, the Collaborative has initially focused on rural-urban geodisparity based on ZIP codes [21], with a longer-term plan to extract residential address and calculate neighborhood disadvantage. The Collaborative staff worked with health systems to submit complete five-digit ZIP codes to geocode to rural-urban geodisparity categories (available at https://www.hipxchange.org/RuralUrbanGroups). The geocoded data will be attached to regular reports sent back to health systems to aid in targeting quality improvement interventions. By centralizing the data processing for construction of geodisparity, the burden on health systems will be low, and this process can easily be extended in the future to more complex measures. Because ZIP codes are a national standard, further harmonization was not required.

Neighborhood disadvantage measures offer improved information on the socioeconomic conditions of a smaller (e.g., census tract) area, but are highly dependent on precise geolocation information. Billing address information is available in all the health systems’ EHRs but the Collaborative determined that very few of the health systems collected separate information on residential address. However, in one study, 89% of billing addresses represented residential addresses [23]. To obtain more precise geolocation information, the Collaborative has requested that health systems begin submitting billing address as part of their transition to a cloud-based data analytics platform. Because many health systems lack the internal geocoding capacity for addresses that is required to construct more complex measures of neighborhood disadvantage, the Collaborative staff will geocode billing address to census tract and nine-digit ZIP code to quantify neighborhood disadvantage using an established index [24]. The geocoded and indexed data will also be sent back to health systems. In addition, health systems will be informed of the importance of residential address and encouraged to develop strategies to collect this information for future improvements to the data repository.

### Missing Data

Our results indicate that high-quality information on disparity indicators can be achieved across a range of health systems from EHR data. For race/ethnicity, two of 25 systems had substantial missing information. System B had 19.5% missing and system Q had 100% missing (Supplementary Table 1). For insurance status and type, six systems had substantial missing, with two having >75% missing (Supplementary Table 2). The amount of missing by insurance status and type is primarily due to mapping issues and not true missing data. For geographic disparity, two of 25 systems had substantial missing (Supplementary Table 3). System A had 18.3% missing and system B had 22.2% missing.

### Disparity Reports

Although not the focus of this manuscript, the successful results of this statewide effort to measure disparities in care have been reported elsewhere [25,26]. Briefly, substantial disparities were identified for American Indian/Alaska Native and Black populations across six measures, including childhood vaccinations, breast cancer screening, and tobacco-free in diabetes and in heart disease. Asian/Pacific Islander, Hispanic/Latino, and White populations experienced substantial disparities for two measures each. Uninsured and Medicaid populations experienced substantial disparities across multiple measures, twelve in total, including childhood vaccinations, blood sugar control in diabetes, recommended weight, cancer, and depression screenings. Commercial and Medicare populations experienced disparities for one measure each. The project’s final statewide report on health disparities is available at www.hipxchange.org/WCHQDisparities.

## Discussion

Public reporting on disparities in the quality of care is critical to achieving accountability for reducing disparities over time [4]. Addressing fundamental upstream causes of disparities such as the social determinants of health is essential to disparities reduction, but is not sufficient [27]. Downstream interventions within health systems are also necessary to address the consequences of these fundamental causes, as poor healthcare quality can create additional disparities in health outcomes [27]. As a result, challenges in the use of EHR data have profound implications for the measurement of disparities in clinical care, reporting, research, and public health [8]. Missing data and data harmonization are key challenges in EHR data. Our experience illustrates the complexities of large-scale health disparities measurement when bringing together EHR data from multiple health systems into a single repository. Building on a statewide, centralized data repository of validated quality measures, we describe a process to select, assess, harmonize, standardize, and implement health disparities indicators to stratify and publicly report the quality measures [25,26]. Disparity indicators include race and ethnicity, insurance status and type, and geographic disparity. We identified substantial challenges to collecting these disparity indicators (e.g., missing data, incomplete data) but identified acceptable solutions for these challenges and in most cases were able to maintain our goal of minimizing burden on the health system. The solutions encompassed health system harmonization for insurance status and type, central harmonization of race and ethnicity, and centralized data processing for geographic disparity.

Health systems that measure and report on the quality of care have rarely focused on health disparity measures. Reported barriers include a lack of standardization of health disparity measures across health systems, lack of race and ethnicity data, data quality, inability to aggregate data from multiple health systems, resource constraints, and competing priorities [9]. In some cases, there was a lack of buy-in due to low perceived diversity within a service area. To maximize our chances of success, we addressed each of these barriers in our approach. We engaged the Collaborative health systems in approving the development of health disparity measures and in selecting the specific disparity indicators using the same process that is used successfully in the development of any new Collaborative quality measure. This addressed issues of standardization, interoperability, and technical challenges and facilitated the allocation of needed resources because the process was known to the data submitters. Engaging health systems in the process facilitates participation and is critical for others seeking to implement similar programs.

The process of assessing disparity indicator quality and developing an implementation plan across 25 health systems was challenging and required substantial communication with each health system’s data submitter. We made significant efforts to reduce the burden on health systems to avoid contributing to more than $15 billion that systems spend annually to report quality measures [28]. There are many known issues in bringing together EHR data from multiple health systems including data quality, completeness, sharing, and transmission issues; organizational structure, maturity, sustainability issues; and vendor issues [12]. The Collaborative has previously and successfully addressed many of these challenges in the construction of quality metrics through a common data model, validation process that involves the frequent review of Collaborative data submission procedures to ensure data completeness, the convening of a standing Collaborative committee to oversee data submission and measure specification development, hands-on support to health systems in their data submission activities, and a semi-annual review of measure results to identify outliers that may be caused by data submission errors. Over the years, strong relationships have been built with data submitters at each health system, which facilitated both the quality assessment process and the development of solutions to challenges with health disparities indicators. These relationships are critical to building and maintaining high-quality data submissions.

### Limitations and Future Directions

Two limitations of this study were missing data and problematic harmonization of data. To address data quality issues for disparity indicators, the Collaborative worked with health systems to clean the race/ethnicity, insurance payer and type, and geographic fields as much as possible, but some health systems did not have data on all of their patients. For example, the missing data for the race/ethnicity fields are a result of patients not being asked and/or patients declining to answer. The Collaborative provided training materials on best practices for asking about disparity indicators and explaining to patients why a health system would want to collect this information, but some health systems still had high missing rates in their EHRs. Future directions to improve missing EHR race and ethnicity data could use natural language processing to identify patient demographic characteristics [29] or have patients directly record their race and ethnicity [30]. For the insurance payer and type field, the Collaborative concluded the amount of missing was due to harmonization issues and not true missing data. To improve the insurance payer and type field, the Collaborative will continue to work with health systems to improve how they assign their payers to the categories in the Collaborative data submission process.

The Collaborative’s common data model is well established, but since 2003 multiple national standard data models have now been developed (e.g., PCORNet CDM, OMOP). Transitioning to a national standard model has been discussed due to the challenges associated with coordinating data extraction/submission from health systems, but to date the burden on health systems of transitioning to a new data submission process would preclude this transition.

## Conclusions

In conclusion, the Collaborative developed a robust and successful implementation plan for public reporting of disparities in healthcare quality at a statewide level. This is a critical prerequisite for achieving accountability for reducing disparities in healthcare quality over time. Based on this work, the Collaborative has launched a statewide disparities improvement team to reduce disparities in colorectal cancer screening in underserved rural areas and blood pressure and A1c control in urban areas. Our process identified and addressed multiple challenges through a flexible approach that minimized burden on the participating health systems. Key lessons learned to support other large-scale collaboratives in incorporating measures of health disparities include engaging health systems in identifying which indicators of health disparity are of interest and aligning with system priorities, measuring health disparity indicators that are already collected in the EHR to minimize the burden on health systems, and facilitating workgroups with health systems to build relationships, improve data collection, and develop initiatives to address disparities in healthcare quality.

## Supplementary material

For supplementary material accompanying this paper visit https://doi.org/10.1017/cts.2022.521. click here to view supplementary material
