# Supplementary material for: Insights into measuring health disparities using electronic health records from a statewide network of health systems: A case study
Source: J Clin Transl Sci. 2023 Feb 1;7(1):e54. doi: 10.1017/cts.2022.521 (PMC10052445; doi:10.1017/cts.2022.521)
Supplement: Supplementary file 1 [file S2059866122005210sup001.docx]

| **Supplemental Table 1. Percent of missing and non-missing information on race/ethnicity for each health system** | | | | | | |
| --- | --- | --- | --- | --- | --- | --- |
| **Health System** | **Missing (%)** | **American Indian/Alaska Native (%)** | **Asian/Pacific Islander (%)** | **Black (%)** | **Hispanic/ Latino (%)** | **White (%)** |
| A | 0.2 | 0.1 | 0.4 | 0.7 | 1.7 | 96.7 |
| B | 19.5 | 0.1 | 0.7 | 15.4 | 2.3 | 42.5 |
| C | 1.3 | 0.2 | 1.2 | 17.6 | 5.4 | 74.3 |
| D | 0.3 | 0.2 | 1.0 | 0.8 | 1.1 | 96.7 |
| E | 0.3 | 0.4 | 1.0 | 0.4 | 0.4 | 97.6 |
| F | 2.8 | 0.0 | 2.0 | 2.7 | 1.0 | 88.7 |
| G | 0.3 | 0.3 | 1.3 | 10.1 | 3.8 | 83.9 |
| H | 1.1 | 0.3 | 0.4 | 1.1 | 1.5 | 94.6 |
| I | 5.3 | 0.2 | 1.1 | 13.8 | 1.0 | 73.5 |
| J | 0.1 | 0.3 | 0.7 | 0.7 | 0.7 | 97.7 |
| K | 1.1 | 0.4 | 1.0 | 0.3 | 1.2 | 96.1 |
| L | 1.9 | 0.2 | 0.6 | 0.8 | 0.8 | 93.9 |
| M | 0.8 | 0.1 | 0.7 | 0.5 | 0.8 | 96.4 |
| N | 1.0 | 0.1 | 0.7 | 7.4 | 3.8 | 85.9 |
| O | 1.0 | 0.2 | 1.5 | 5.2 | 2.1 | 89.1 |
| P | 0.4 | 0.2 | 0.2 | 4.0 | 1.0 | 94.2 |
| Q | 100.0 | 0.0 | 0.0 | 0.0 | 0.0 | 0.0 |
| R | 0.9 | 0.0 | 0.8 | 1.0 | 1.1 | 95.3 |
| S | 0.4 | 0.1 | 0.9 | 0.5 | 0.5 | 97.3 |
| T | 1.6 | 0.1 | 0.7 | 0.7 | 1.5 | 93.8 |
| U | 1.5 | 0.5 | 0.4 | 0.2 | 1.0 | 94.8 |
| V | 0.6 | 0.4 | 0.7 | 0.6 | 1.1 | 96.1 |
| W | 0.8 | 0.3 | 2.1 | 4.2 | 1.7 | 90.8 |
| X | 0.8 | 0.1 | 0.2 | 0.7 | 0.3 | 97.2 |
| Y | 1.2 | 0.1 | 0.5 | 1.4 | 0.0 | 95.7 |

# SUPPLEMENTARY MATERIAL

| **Supplemental Table 2. Percent of missing and non-missing information on insurance status and type for each health system** | | | | | |
| --- | --- | --- | --- | --- | --- |
| **Health System** | **Missing (%)** | **Commercial (%)** | **Medicare (%)** | **Medicaid (%)** | **Uninsured (%)** |
| A | 42.6 | 0.5 | 56.9 | 0.0 | 0.0 |
| B | 0.0 | 38.8 | 54.6 | 5.4 | 1.2 |
| C | 0.1 | 36.3 | 52.9 | 9.3 | 1.4 |
| D | 4.3 | 38.0 | 52.7 | 4.2 | 0.9 |
| E | 0.2 | 56.2 | 32.3 | 5.3 | 5.9 |
| F | 65.9 | 17.0 | 16.2 | 0.6 | 0.0 |
| G | 0.0 | 67.9 | 25.8 | 5.0 | 1.3 |
| H | 0.0 | 66.9 | 26.2 | 5.1 | 1.8 |
| I | 16.1 | 28.9 | 52.0 | 3.0 | 0.0 |
| J | 0.0 | 38.7 | 54.3 | 4.4 | 2.7 |
| K | 0.0 | 34.1 | 60.3 | 4.9 | 0.7 |
| L | 1.0 | 63.4 | 26.6 | 9.0 | 0.0 |
| M | 1.3 | 46.4 | 46.0 | 6.3 | 0.0 |
| N | 11.5 | 35.5 | 46.6 | 5.6 | 0.8 |
| O | 78.8 | 10.9 | 9.4 | 0.8 | 0.1 |
| P | 1.9 | 45.8 | 48.3 | 2.7 | 1.3 |
| Q | 100.0 | 0.0 | 0.0 | 0.0 | 0.0 |
| R | 8.0 | 33.1 | 55.3 | 3.6 | 0.0 |
| S | 0.0 | 79.6 | 18.6 | 1.3 | 0.6 |
| T | 0.0 | 37.7 | 30.2 | 2.7 | 29.4 |
| U | 0.9 | 35.5 | 58.6 | 4.2 | 0.9 |
| V | 0.0 | 75.9 | 19.5 | 3.5 | 1.2 |
| W | 3.4 | 42.7 | 50.7 | 3.2 | 0.0 |
| X | 0.0 | 42.3 | 53.0 | 2.9 | 1.8 |
| Y | 0.0 | 85.5 | 12.1 | 1.8 | 0.6 |

| **Supplemental Table 3. Percent of missing and non-missing information on ZIP code for each health system** | | | |
| --- | --- | --- | --- |
| **Health System** | **Missing (%)** | **Rural (%)** | **Urban (%)** |
|  |  |  |  |
| A | 18.3 | 7.5 | 74.2 |
| B | 22.2 | 0.4 | 77.4 |
| C | 0.0 | 0.5 | 99.5 |
| D | 0.0 | 46.4 | 53.6 |
| E | 0.0 | 57.2 | 42.8 |
| F | 0.0 | 3.2 | 96.8 |
| G | 0.0 | 10.3 | 89.7 |
| H | 0.0 | 41.9 | 58.1 |
| I | 1.6 | 1.2 | 97.2 |
| J | 0.0 | 41.4 | 58.6 |
| K | 0.0 | 62.7 | 37.3 |
| L | 0.0 | 35.5 | 64.6 |
| M | 0.0 | 22.5 | 77.5 |
| N | 0.0 | 12.1 | 87.9 |
| O | 0.0 | 4.7 | 95.3 |
| P | 0.0 | 84.5 | 15.5 |
| Q | 0.0 | 30.2 | 69.8 |
| R | 0.0 | 11.5 | 88.5 |
| S | 0.1 | 4.9 | 95.0 |
| T | 0.0 | 2.4 | 97.6 |
| U | 0.1 | 89.1 | 10.9 |
| V | 0.1 | 37.4 | 62.5 |
| W | 0.1 | 15.5 | 84.4 |
| X | 0.0 | 6.3 | 93.7 |
| Y | 0.0 | 2.9 | 97.1 |
